# Amelioration of testicular damages in renal ischemia/reperfusion by berberine: An experimental study

**DOI:** 10.18502/ijrm.v17i10.5488

**Published:** 2019-11-28

**Authors:** Firouzeh Gholampour, Shabnam Malekpour Mansourkhani, Seyed Mohammad Owji

**Affiliations:** ^1^Department of Biology, School of Sciences, Shiraz University, Shiraz, Iran.; ^2^Department of Pathology, School of Medicine, Shiraz University of Medical Sciences, Shiraz, Iran.

**Keywords:** Ischemia/reperfusion, Acute kidney injury, Berberine, Testis, LH, FSH.

## Abstract

**Background:**

Ischemic acute kidney injury is associated with an inflammatory reaction.

**Objective:**

In the current study, berberine was assessed for its effect on the functional disorders and histological damages of testis induced by renal ischemia/reperfusion (I/R).

**Materials and Methods:**

Twenty-eight adult male Wistar rats (260-300 gr) were equally divided into four groups (n = 7/each): sham and I/R groups which received distilled water as well as berberine (BBR) and BBR + I/R groups which received berberine (15 mg/kg/day) orally seven days before the surgery. In both groups of sham and BBR, renal arteries were not clamped. Renal I/R was induced by occluding right and left renal artery for 45 min followed by a 24 hr reperfusion period. Blood samples were taken for determining the plasma levels of creatinine, urea nitrogen, FSH (follicle stimulating hormone), LH (luteinizing hormone), and testosterone. Then the rats were killed under deep anesthesia and the left testis was immediately isolated and preserved.

**Results:**

The renal I/R injury led to testicular histological damages accompanied with increased plasma levels of creatinine, urea nitrogen, LH, and FSH, as well decrease of plasma testosterone concentration at the end of 24 hr reperfusion (All p < 0.001, except for FSH p < 0.01). Berberine diminished histological damage to the testis and attenuated the increase in plasma creatinine, urea nitrogen, LH, FSH, and decrease in plasma testosterone concentration in the BBR + I/R group (All p < 0.001).

**Conclusion:**

These results suggest that ischemic acute renal failure induces functional disorders and tissue damages in testis of rat, which was improved through the administration of berberine.

## 1. Introduction

In the ischemic acute kidney injury (AKI), the inflammatory reaction results in endothelial injury, augmented endothelial cell-leukocyte adhesion, trapping of leukocytes, and compromised vascular blood flow. Besides, following ischemic injury, renal tubular epithelial cells potentiate inflammation by generating pro-inflammatory cytokines (1). It has been suggested that an enormous influx of neutrophils causes release of cytotoxic proteases and oxygen reactive species and thereby leads to post-ischemic renal failure (2). Moreover, it is believed that AKI creates injury to distant organs such as heart, brain, liver, and lungs via mechanism that involves oxidative stress, neutrophil migration, and increased cytokine concentrations (3). Acute stresses are associated with gonadotropin suppression and cause harm to testicular function (4). It has been reported that renal failure is responsible for testicular dysfunction (5). In this regard, chronic renal failure impairs to spermatogenesis and steroidogenesis by affecting all levels of the hypothalamic-pituitary-testicular axis. Perturbations of the axis can be detected with just modest decreases of the glomerular filtration rate (GFR) and deteriorate increasingly as the renal failure proceeds (6). Berberine, an isoquinoline alkaloid derived from stem bulk, roots, and fruits of the plants such as *Berberis Vulgaris* have anti-inflammatory (7), antioxidant (8), and antibacterial (9) activities.

Considering the potential therapeutic properties of berberine, the goals of this experiment were to determine the effects of AKI on the testicular function and to assess the effect of berberine on testicular damages induced by renal I/R injury.

## 2. Materials and Methods

### Experimental procedure

Twenty-eight male Wistar rats weighing 260-300 gr were obtained from Razi institute (Shiraz, Iran). They were caged under standard laboratory conditions at 24-26°C for 12-hr light/dark cycles and had free access to food and water ad libitum. In this experimental study, the ethics of working with an animal has been respected. The animals were randomly and equally divided into four groups: Sham, BBR (Berberine, 15 mg/kg/day for seven days), I/R, BBR + I/R (Berberine, 15 mg/kg/day for seven days). After seven days of gavage administration of distilled water/berberine, anesthesia was performed by intraperitoneal injection of 60 mg/kg ketamine and 5 mg/kg xylazine. Anesthetized rats were subjected to median laparotomy on a heated surgical table, which kept the animal warm at 37 ± 1°C. Thereafter, under the surgical microscope, the right and left renal arteries were cautiously separated from the right and left renal veins. In the sham and BBR groups, renal arteries were not clamped and daily administrations of distilled water and barberine, respectively, were made for seven days before the surgery. However, in the I/R and BBR + I/R groups, rats were subjected to 45 min ischemia by clamping of both the renal arteries. Then, they received distilled water and berberine, respectively, for seven days before the surgery. At the end of 24-hr reperfusion, blood samples were collected from heart ventricles under anesthesia. Then the rats were killed under deep anesthesia and the left testis was immediately isolated, weighed, and preserved.

### Biochemical tests

Plasma samples were evaluated for urea nitrogen and creatinine in milligram per deciliter by colorimetric methods (autoanalyzer; Prestige, Biolis 24I, Japan). Plasma levels of testosterone, luteinizing hormone (LH), and follicle stimulating hormone (FSH) were assayed using rat testosterone, LH and FSH kits (Institute of Isotopes Co., Ltd, Budapest, Hungary), and the method of radioimmunoassay. The sensitivity for testosterone, LH and, FSH kits were 0.05, 0.05, and 0.04 ng/ml.

### Histopathological evaluation

The left testis samples were fixed in formalin (10% phosphate-buffered). After dehydration in different grades of alcohol, the samples were cleared in xylol and were embedded in paraffin. Then, 5-μm-thick sections were stained with H&E. Histopathological scoring for each testicular section was performed blindly using a light microscope in at least 10 randomly selected non-overlapping fields. The testicular histopathology was evaluated for the atrophy of seminiferous tubules, Leydig cell hyperplasia, and increase in interstitial connective tissue. All histopathological manifestations were scored (10) pursuant to the percentage of testis tissue involved as follow: 0 (absent), 1 (less than 20%), 2 (21-40%), 3 (41-60%), 4 (61-80%), and 5 (more than 80%). In each group, the sum of all numerical scores was considered as the total histopathological score (Table I).

**Table 1:** The total histopathological score in groups at the end of 24 hr reperfusion


**Experimental groups**	**Testis**
Sham	0.0 ± 0.00
BBR	0.0 ± 0.00
I/R	19.42 ± 2.29${}^{***}$
BBR + I/R	9.85 ± 1.21${}^{†††}$
Values (sum of histopathological scores in each group) are expressed as Mean ± SD	
${}^{***}$P < 0.001 as compared to the sham and BBR; ${}^{†††}$P < 0.001 as compared to the I/R
BBR: Berberine; I/R: Ischemia/Reperfusion; BBR+ I/R: Berberine + Ischemia/Reperfusion

### Ethical consideration

All interventions in rats were provided according to the protocol provided by the ethics committee of Shiraz University (code: 900794).

### Statistical analysis

Experimental data (mean ± SD) were analyzed using the SPSS statistics software package (SPSS for Windows version 16, Chicago, IL, USA). The normal distribution was tested by Shapiro-Wilk. Comparison between groups was calculated by one-way ANOVA and Tukey's post-hoc test. The histopathological scores were statistically compared between groups by Kruskal-Wallis multiple comparison tests, and binary comparisons were done by Mann-Whitney U test. P < 0.05 was considered significant.

## 3. Results

### Berberine ameliorates the I/R-induced changes in biochemical parameters

**Figure 1:**
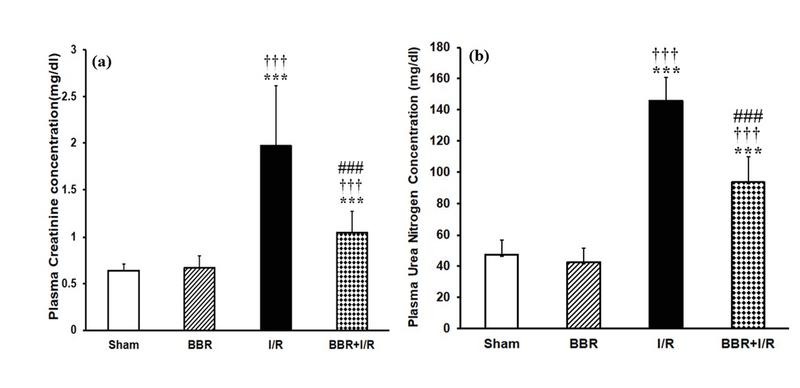
Berberine affects plasma levels of (a) creatinine and (b) urea nitrogen. ***P < 0.001 vs sham group; ${}^{†††}$P < 0.001 vs BBR group; ${}^{♯♯♯}$P < 0.001 vs I/R group.

**Figure 2:**
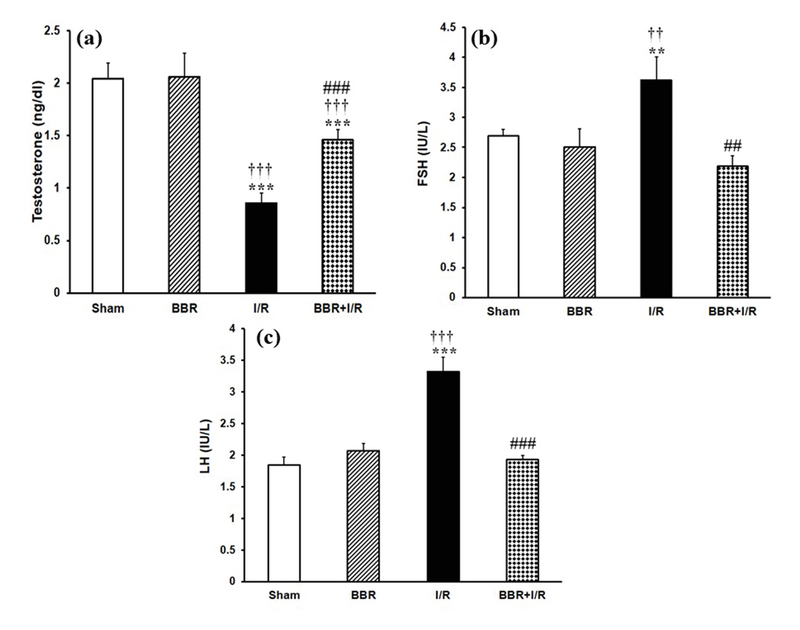
Berberine affects plasma levels of (a) testosterone, (b) LH, and (c) FSH. **P < 0.01, ***P < 0.001 vs sham group; ${}^{††}$P < 0.01, ${}^{†††}$P < 0.001 vs BBR group; ${}^{♯♯}$P < 0.01, ${}^{♯♯♯}$P < 0.001 vs I/R group.

As shown in Figure 1, at the end of 24 hr reperfusion period, the plasma levels of creatinine and urea nitrogen were statistically higher in I/R group in comparison with those of sham and BBR groups (p < 0.001). However, in the BBR + I/R group, there was a significant decrease in creatinine (Figure 1a) and BUN (Figure 1b) levels compared to the I/R group (p < 0.001). Figure 2a shows that in the I/R group, the plasma testosterone level was significantly decreased with respect to its level in the sham and BBR groups (p < 0.001). The testosterone level was increased in the BBR + I/R group in comparison to the I/R group (p < 0.001), while it was lower than the level in the sham and BBR groups (Figure 2a). Besides, plasma levels of LH and FSH observed were significantly increased in the I/R group compared to the sham and BBR groups (p < 0.001). But berberine pretreatment diminished the plasma levels of LH (Figure 2b) and FSH (Figure 2c) in the BBR + I/R group in comparison with the I/R group (p < 0.001).

### Berberine protects the I/R-induced histological changes in testicular tissue

**Figure 3:**
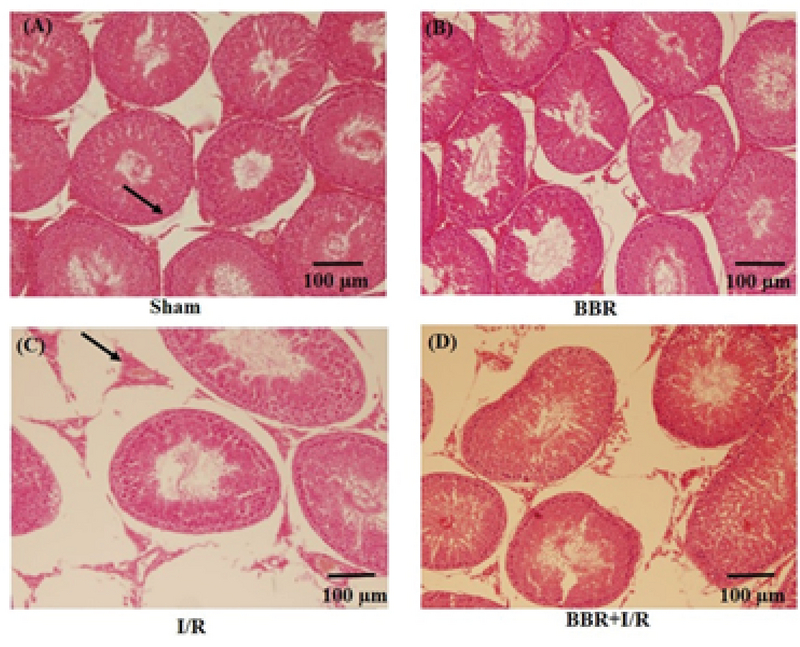
Representative light microphotographs of the testis in the (A) sham, (B) BBR, (C) I/R, and (D) BBR + I/R groups. Testicular sections stained with H&E (magnification × 100) were assessed for histological changes at the end of 24-hr reperfusion including increased volume of interstitial space (black arrows).

**Figure 4:**
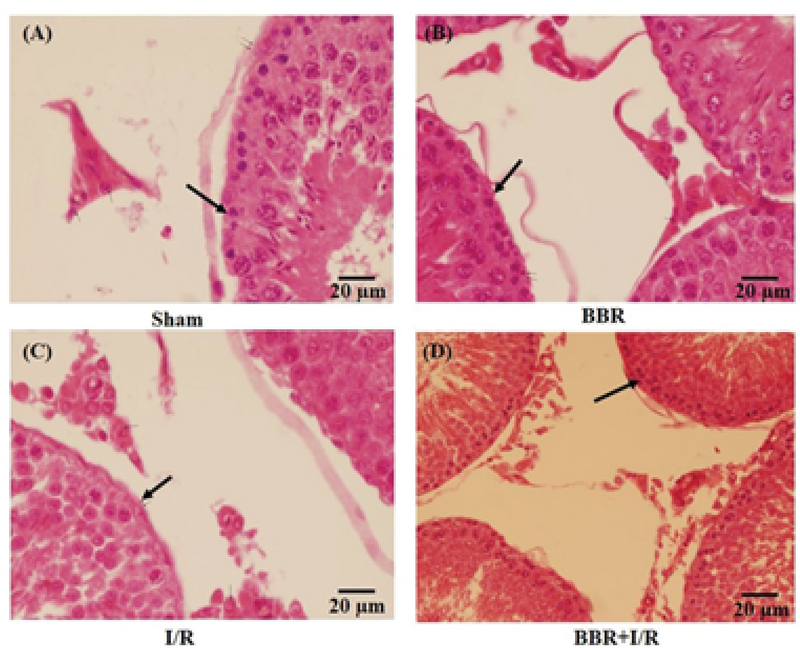
Representative light microphotographs of the testis in the (A) sham, (B) BBR, (C) I/R, and (D) BBR + I/R groups. Testicular sections stained with H&E (magnification × 400) were assessed for histological changes at the end of 24-hr reperfusion including the thickening of the seminiferous tubular basement membrane (black arrows).

**Figure 5:**
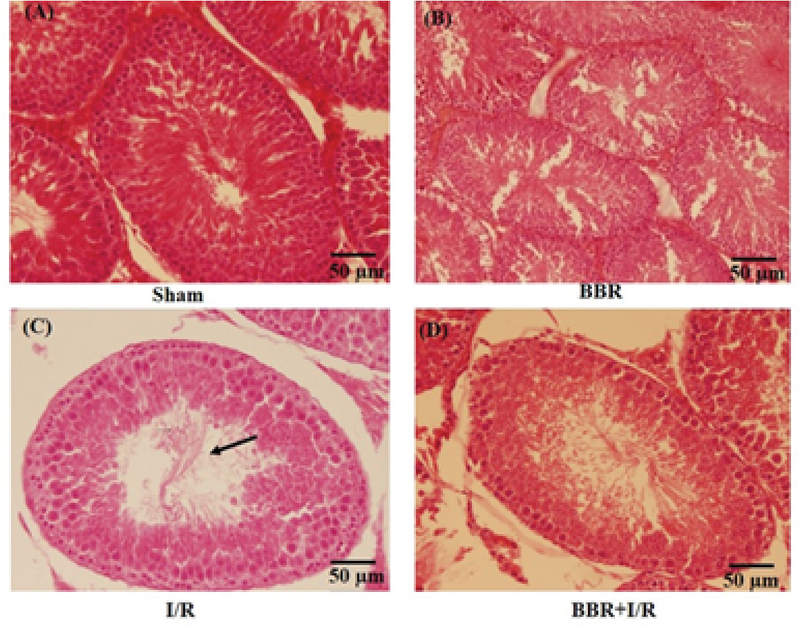
Representative light microphotographs of the testis in the (A) sham, (B) BBR, (C) I/R, and (D) BBR + I/R groups. Testicular sections stained with H&E (magnification × 200) were assessed for histological changes at the end of 24-hr reperfusion including reduced percent of spermatogenesis (black arrows).

Figures 3, 4, and 5 shows that there were no detectable histological changes to the testes of the sham and BBR groups. However, the most prominent lesions in the I/R group (Figure 3, 4, 5) were increased number of interstitial Leydig cells (hyperplasia), increase in the volume percent of interstitial space and internal space of seminiferous tubules, decrease in the number of spermatogenic cells, and thickening of the tubular basement membrane. In the BBR + I/R group, less intense damages were noticed in comparison with the I/R group (Figures 3, 4, 5).

## 4. Discussion

The AKI is assumed as a pan-organ problem with negative consequences on many other organs in the body (11). The main goal of this study was to evaluate the effect of 45 min ischemia/24 hr reperfusion on functional-morphological characteristics of the testis in rats. The reason for considering 45 min ischemia/24 hr reperfusion as a timing of ischemia and reperfusion was that a minimum of 30 min ischemia is needed to induce AKI. Besides, mitochondrial function is restored by reperfusion when ischemic interval is 45 min or less. However, ischemic intervals longer than 45 min produce non-reversible impairment of ATP synthesis, and the marked reduction following 90 min of ischemia signifies possible transition to a non-viable state (12). Also, the maximum renal dysfunction following 30-45 min of renal ischemia happens at the end of 24 hr reperfusion period (13). The renal IRI resulted in testicular histological damages accompanied with increased plasma levels of creatinine, urea nitrogen, LH, and FSH, as well decrease of plasma testosterone concentration. Berberine diminished histological damage to the testis and attenuated the increase in plasma creatinine, urea nitrogen, LH, FSH, and decrease in plasma testosterone concentration in the BBR + I/R group.

In sham rats, the seminiferous tubules had normal size and were full of spermatogenic cells, with minimal interstitial connective tissue and a small number of Leydig cells. Morphological study of the testicular sections in the I/R group revealed a decreased number of spermatogenic cells, thickening of basement membrane, and Leydig cell hyperplasia. As very high quantities of testosterone are essential for the survival of the reproductive tract, it is concluded that this atrophy of seminiferous tubules is probably developed due to the reduction in the testosterone levels. Besides, Leydig cell hyperplasia might be created to offset the reduced testosterone level.

Our results showed elevated plasma LH concentration after the induction of I/R injury (Figure 2b). This can be due to the diminished testosterone feedback. The significant reduction in plasma testosterone level in the I/R group has been observed in the other investigations too (14). Another reason for the elevation in the LH levels is probably insufficient renal excretion. In agreement with our results, a decrease in the testosterone serum level concomitant to the elevated LH levels in patients who suffer from chronic renal failure has been reported previously (15, 16). An interpretation for the significant reduction in plasma testosterone level in the I/R group, despite the elevated LH levels, is that the response of testis to the LH tends to be reduced. In this regard, there is evidence of the existence of a factor in uremic serum which can block the LH receptor and is inversely correlated with the GFR (17). The reduced circulating LH bioactivity had been reported in chronic kidney failure (18). Moreover, the elevation in the FSH levels is probably due to decreased inhibin, which is produced by the Sertoli cells (16). Recently, a dysfunction of Sertoli cell was reported in the men suffering from an end-stage renal disease (19). FSH stimulates spermatogenesis. Histopathological study of the testis in the I/R group showed some disturbances in the seminiferous tubules, including the thickening of the basement membrane of the tubules, increased interstitial space, and decreased number of spermatogenic cells, particularly the number of spermatogonia. Testosterone, as a steroid hormone, is needed for spermatogenesis (20). Antioxidants can protect the cells and tissues against the detrimental effects of free radicals (21). The administration of berberine to the I/R group significantly augmented the level of testosterone. The spermatogenic inhibition observed in the I/R group in the current work cannot merely be caused due to the reduction in the plasma testosterone level. In addition to the hormonal change, the spermatogenic inhibition may also be created consequently to the establishment of free radicals in the testis due to their harmful effect on spermatogenesis. The improvement in the spermatogenesis observed in the BBR + I/R group may be associated with the antioxidant property of the berberine. Berberine is described by a variety of pharmacological effects including antioxidant and anti-inflammatory properties (22). In agreement with our results, berberine improved the testosterone level in the conditions of testicular inflammation and oxidative stress induced by gossypol (23). In the current research, the administration of berberine (15 mg/kg) for seven serial days significantly diminished the I/R-induced increase in the plasma levels of LH and FSH hormones. This decrease of LH and FSH levels could be due to the ameliorative effect of berberine on the function of both kidney and testis. The improvement in the renal function, as indicated by a reduction in I/R-induced increment in plasma levels of Cr and BUN, led to sufficient excretion of LH. Besides, the decrease of renal I/R-induced testicular damages by berberine resulted in improved function of sertoli cells. Consequently, plasma FSH level returned to normalcy due to sufficient inhibition by inhibin.

## 5. Conclusion

In summary, 45-min renal ischemia/24-hr reperfusion caused renal and testicular dysfunction. It also induced disturbances in the pituitary-gonadal axis. Gavage administration of berberine prior to the induction of renal I/R injury blunted functional disorders of kidney and testis and consequently diminished the increases in plasma levels of creatinine, urea nitrogen, LH and FSH, as well as a decrease in plasma level of testosterone. Our findings propose that berberine has the potential therapeutic property for the amelioration of testicular damages induced by renal I/R injury due to its antioxidant and anti-inflammatory properties.

##  Conflict of Interest

The authors of this article declare that they have no conflict of interest.
